# Hidden Challenges in Laparoscopic Instrument Maintenance: A Qualitative Exploration of Training and Safety Concerns

**DOI:** 10.7759/cureus.112734

**Published:** 2026-07-15

**Authors:** Amruta Choudhary, Anusha Kamath, Neha Gangane, Priyanka Yadav, Avinash Prakash, Anita Yadav, Nitin Marathe

**Affiliations:** 1 Obstetrics and Gynaecology, All India Institute of Medical Sciences, Nagpur, Nagpur, IND; 2 Community Medicine, All India Institute of Medical Sciences, Nagpur, Nagpur, IND; 3 Anaesthesiology, All India Institute of Medical Sciences, Nagpur, Nagpur, IND; 4 Hospital Administration, All India Institute of Medical Sciences, Nagpur, Nagpur, IND

**Keywords:** instrument maintenance, quality improvement, simulation-based training, sterilization, troubleshooting

## Abstract

Background: Over the past two decades, the rapid expansion of laparoscopic procedures has substantially increased dependence on sophisticated surgical instruments, camera systems, insufflators, energy devices such as monopolar and bipolar electrocautery, advanced bipolar and ultrasonic devices, and advanced sterilization technologies. Consequently, the success of laparoscopic surgery depends not only on surgical expertise but also on the effective functioning, maintenance, reprocessing, and handling of specialized equipment by the operating theater (OT) team. Aim: The study aimed to qualitatively explore the experiences, operational difficulties, safety concerns, and training needs related to laparoscopic instrument maintenance among OT technicians, nursing officers, and residents in the Department of Obstetrics and Gynecology of a tertiary care teaching hospital. Materials and methods: A descriptive qualitative study was conducted in the Department of Obstetrics and Gynecology at the tertiary care teaching hospital. The study participants included residents, nursing officers, and OT technicians who were directly involved in laparoscopic surgeries and perioperative instrument management. A quota-based purposive sampling technique was used to ensure adequate representation, and a total of 30 participants were included, comprising 10 residents, 10 nursing officers, and 10 OT technicians. Focus group discussions (FGDs) were selected as the data collection method. The study was conducted and reported in accordance with the Consolidated Criteria for Reporting Qualitative Research (COREQ) guidelines. Data were collected using a semi-structured interview and continued until thematic saturation was achieved. Results: Thematic analysis of the FGDs identified six major themes related to laparoscopic instrument maintenance and handling practices: training deficiencies, equipment-related challenges, sterilization and reprocessing issues, instrument-handling difficulties, safety concerns, and workflow and communication barriers. OT technicians predominantly discussed technical and maintenance-related concerns, including damage to delicate cables, preventive servicing requirements, and sterilization limitations. Nursing officers mainly emphasized workflow coordination, patient safety concerns, and the need for standardized checklists. Residents primarily highlighted deficiencies in troubleshooting skills and formal laparoscopic training. Conclusions: The present study highlights that laparoscopic instrument maintenance is an important yet often underrecognized component of minimally invasive surgical safety.

## Introduction

Laparoscopic surgery has revolutionized modern surgical practice by offering minimally invasive approaches associated with reduced postoperative pain, shorter hospital stays, faster recovery, and improved cosmetic outcomes compared with conventional open procedures. However, these benefits depend on surgical expertise, patient selection, and available technology [1]. Over the past two decades, the rapid expansion of laparoscopic procedures has substantially increased dependence on sophisticated surgical instruments, camera systems, insufflators, energy devices (such as monopolar/bipolar electrocautery and ultrasonic shears), and advanced sterilization technologies. Consequently, the success of laparoscopic surgery depends not only on surgical expertise but also on the effective functioning, maintenance, reprocessing, and handling of specialized equipment by the operating theater (OT) team.

Laparoscopic instruments are delicate, expensive, and technically complex devices that require meticulous cleaning, disinfection, sterilization, and preventive maintenance to ensure patient safety and prolong instrument lifespan [2]. Improper reprocessing and reuse of disposable accessories, often driven by resource limitations, may result in equipment malfunction, gas leakage, poor visualization, operative delays, and increased risk of infection transmission [3]. In low- and middle-income healthcare settings, these challenges are further compounded by inadequate infrastructure, shortage of trained personnel, limited access to preventive maintenance services, and lack of standardized reprocessing protocols [4].

OT technicians, nursing officers, and surgical residents constitute the frontline workforce responsible for laparoscopic equipment preparation, troubleshooting, sterilization, and intraoperative support. Previous studies have demonstrated that deficiencies in training, workflow interruptions, ergonomic burden, and communication gaps within the OT can adversely affect surgical efficiency and patient safety [5,6]. Furthermore, OT nurses have reported significant challenges in maintaining sterile technique and operating technologically advanced surgical systems, underscoring the need for continuous competency-based training and multidisciplinary coordination [7].

Although previous literature has explored technical and educational aspects of laparoscopic surgery, most studies have focused primarily on surgical outcomes, operative techniques, or surgeon-centered training. Qualitative evidence examining the practical experiences of OT personnel involved in laparoscopic instrument maintenance, sterilization, troubleshooting, and perioperative workflow management remains limited, particularly in the context of minimally invasive gynecological surgery in Indian tertiary-care hospitals [8].

Understanding the perspectives of OT technicians, nursing officers, and residents is essential to identifying system-level gaps that affect laparoscopic safety practices and operational efficiency. Such insights may contribute to the development of institution-based maintenance protocols, standardized sterilization practices, simulation-based training modules, and quality improvement initiatives to strengthen patient safety in minimally invasive surgery [9].

Therefore, the present study aimed to explore the experiences, operational difficulties, safety concerns, and training needs related to laparoscopic instrument maintenance among OT technicians, nursing officers, and residents in the Department of Obstetrics and Gynecology of a tertiary care teaching hospital.

## Materials and methods

A qualitative descriptive study using focus group discussions (FGDs) was conducted in the Department of Obstetrics and Gynecology at a tertiary care teaching hospital in central India. The study focused on understanding practical issues associated with laparoscopic instrument preparation, sterilization, preventive maintenance, troubleshooting, and intraoperative management in the OT setting. FGDs were selected as the data collection method because they facilitate interactive discussion, encourage the sharing of experiences, and provide deeper insights into operational and safety-related concerns encountered during routine laparoscopic procedures. The study was conducted and reported in accordance with the Consolidated Criteria for Reporting Qualitative Research (COREQ) guidelines to enhance transparency and methodological rigor.

The study participants included residents, nursing officers, and OT technicians who were directly involved in laparoscopic surgeries and perioperative instrument management within the department. A quota-based purposive sampling technique was used to ensure equal representation from each participant category (residents, nursing officers, and OT technicians), as each group has distinct roles in laparoscopic instrument management. A total of 30 participants were included in the study, comprising 10 residents, 10 nursing officers, and 10 OT technicians. Participants were selected based on their active involvement in laparoscopic procedures and willingness to participate in the study. Residents from the Department of Obstetrics and Gynecology involved in laparoscopic surgeries, nursing officers posted in laparoscopic OTs, and OT technicians responsible for laparoscopic equipment and sterilization were included. Individuals unwilling to participate were excluded from the study.

Data were collected using a semi-structured interview guide developed after extensive literature review and expert consultation. The interview guide included open-ended questions designed to explore participants’ experiences regarding laparoscopic instrument handling, maintenance practices, sterilization techniques, equipment-related problems, workflow challenges, communication issues, patient safety concerns, and training requirements. Separate FGDs were conducted for residents, nursing officers, and OT technicians to maintain group homogeneity and minimize hierarchical power differentials, thereby facilitating free expression of opinions. Each focus group consisted of approximately six to eight participants and was conducted in a quiet and confidential environment within the department. Discussions were moderated by the principal investigator, while another investigator documented field observations, contextual details, and non-verbal cues. Each session lasted approximately 45-60 minutes and was audio-recorded after obtaining written informed consent from all participants. Confidentiality and anonymity were assured prior to the discussions, and participants were informed about their right to withdraw from the study at any stage without any consequences.

Data collection continued until thematic saturation was achieved. Saturation was observed after completion of the third FGD, when no substantially new concepts, categories, or perspectives emerged from participant responses. The investigators determined saturation through continuous concurrent analysis of transcripts and repeated review of coding patterns after each discussion. Emerging codes and themes from subsequent FGDs were compared with previously identified categories to assess redundancy and thematic completeness. Although additional FGDs were considered, further data collection was not pursued because subsequent discussions yielded redundant information and failed to generate novel insights relevant to the study objectives.

All audio-recorded discussions were transcribed verbatim immediately after completion of data collection. Responses provided in local languages were translated into English while preserving contextual meaning. The collected qualitative data were analyzed using Braun and Clarke’s six-phase thematic analysis framework, including familiarization, coding, theme generation, theme review, theme definition, and reporting. Initially, transcripts were read repeatedly to become familiar with the data. Meaningful statements and recurring ideas were independently identified and coded by two investigators to improve reliability and minimize subjective bias. Similar codes were grouped into categories and subsequently organized into broader themes: equipment challenges, sterilization practices, workflow barriers, communication gaps, safety concerns, and training deficiencies. An inductive coding approach was followed, allowing themes to emerge naturally from participant responses rather than from predefined assumptions. Discrepancies in coding and interpretation were resolved through discussion and consensus among the investigators.

The credibility and trustworthiness of the findings were ensured through investigator triangulation, repeated transcript review, member checking, and systematic documentation of the analytical process. Reflexivity was maintained throughout the study through regular discussions among investigators about the interpretation of participant responses and potential researcher bias. Ethical approval for the study was obtained from the Institutional Ethics Committee of All India Institute of Medical Sciences, Nagpur (approval number: IEC/Pharmac/2025/1189) prior to the commencement of the study. Written informed consent was obtained from all participants before participation. Confidentiality was maintained by removing personal identifiers during transcription and analysis, and all study-related data were securely stored with access restricted to the research team.

## Results

A total of 30 healthcare professionals participated in the study, including 10 OT technicians, 10 nursing officers, and 10 residents from the Department of Obstetrics and Gynecology. All participants were actively involved in laparoscopic procedures and perioperative instrument management. The mean age of the participants was 29.8 ± 3.6 years, with an age range of 24-38 years. Female participants constituted the majority of the study population (23, 76.7%). The mean professional experience among participants was 3.2 ± 0.8 years. Additionally, 22 (73.3%) of participants had performed more than 50 laparoscopic procedures, while only nine (30.0%) had received prior formal laparoscopic training. Previous troubleshooting experience with laparoscopic equipment was reported by 14 (46.7%) participants. The demographic and professional characteristics of the participants are summarized in Table 1.

**Table 1:** Demographic characteristics of the study participants OT: operating theater, SD: standard deviation

Category	Characteristic	Value (%)
Participant distribution	Total participants	30
	OT technicians, n (%)	10 (33.3)
	Nursing officers, n (%)	10 (33.3)
	Residents, n (%)	10 (33.3)
Age characteristics	Mean age ± SD (years)	29.8 ± 3.6
	Age range (years)	24-38
Gender distribution	Male participants, n (%)	07 (23.3)
	Female participants, n (%)	23 (76.7)
Professional experience	Mean professional experience ± SD (years)	3.2 ± 0.8
Laparoscopic exposure and training	Participants involved in >50 laparoscopic procedures, n (%)	22 (73.3)
	Participants with prior formal laparoscopic training, n (%)	9 (30.0)
	Participants reporting previous troubleshooting experience, n (%)	14 (46.7)

Thematic analysis of the FGDs identified six major themes: training deficiencies, equipment-related challenges, sterilization and reprocessing issues, instrument handling difficulties, safety concerns, and workflow and communication barriers.

Training deficiencies emerged as one of the most prominent concerns across all participant groups. Participants repeatedly highlighted the lack of structured workshops, simulation-based learning opportunities, and formal orientation sessions on laparoscopic instrument handling and troubleshooting. Residents particularly described a lack of confidence in independently assembling laparoscopic towers, connecting insufflator systems, and identifying camera-related problems during emergency procedures.

Before entering the OT, there is very little practical hands-on training. During emergency cases, we often feel helpless and rely on OT technicians to help with equipment setup and troubleshooting. (Resident 4)

At present, we have very limited opportunities for practical hands-on learning, so residents should get simulation-based training before assisting in laparoscopic surgeries. (Resident 2)

We know the surgical steps, but we are not confident about handling equipment failure independently. (Resident 7)

Equipment-related challenges were frequently discussed and included fogging of camera lenses, overheating of light-source cables, malfunctioning insufflator tubing, insufficient availability of monitors, and CO₂ leakage from reused trocars. Participants perceived these technical problems to contribute to procedural delays, surgical interruptions, and increased stress among OT personnel.

We feel very stressful during technical glitches, like when the camera starts fogging or there is CO₂ leakage during surgery; the whole procedure gets delayed, and it creates a lot of stress for everyone in the OT. (Nursing Officer 2)

Even small technical issues create a lot of stress and become difficult to manage during surgery because backup instruments or equipment are not always immediately available in the OT. (OT Technician 8)

Sterilization and reprocessing concerns were identified as major operational and patient safety issues. Participants described challenges related to improper drying of instruments before plasma (STERRAD) sterilization, inadequate cleaning of internal channels, and repeated reuse of disposable laparoscopic accessories due to resource limitations.

We feel vulnerable sometimes as emergency cases often come one after another, there is very little time between procedures, and sometimes instruments may not dry completely before sterilization. (OT Technician 5)

We even feel guilty sometimes as, due to limited availability of supplies, disposable trocars and tubing are sometimes resterilized and used in the OT, even though we know it is not the ideal practice. (Nursing Officer 6)

If instruments are not cleaned properly or moisture remains inside them, sterilization may not be effective, and therefore, we are always under the pressure of risk of surgical infections for patients. (Nursing Officer 3)

Workflow inefficiencies and communication gaps were mainly highlighted by nursing officers and residents. Participants reported delays during emergency laparoscopic setup, absence of standardized preoperative checklists, and inadequate coordination between surgeons and OT personnel.

During emergency laparoscopic cases, communication gaps between the OT team members sometimes delay equipment setup and arrangement of instruments, and this causes a lot of chaos in OT. (Nursing Officer 7)

There is no fixed checklist for preparing laparoscopic equipment, so the setup process often differs from one staff member to another, creating a lot of confusion for us. (OT Technician 6)

Instrument-handling difficulties were reported more frequently among junior residents, who described challenges in identifying laparoscopic instruments and in independently assembling camera systems and insufflators. Many residents expressed dependence on OT technicians during laparoscopic setup procedures and recommended simulation-based training sessions and structured orientation programs for junior trainees.

In the beginning, many laparoscopic instruments appear very similar, and it creates a lot of confusion to identify the correct connectors, cables, and ports during setup. (Resident 3)

Learning how to correct camera alignment and perform white balancing becomes difficult without repeated hands-on practice and regular exposure in the OT. (Resident 1)

Most residents know the procedure, but when it comes to setting up the laparoscopic system in the OT, they feel helpless and usually ask technicians for help because hands-on practice is less during training. (OT Technician 4)

Participants across all groups emphasized concerns about patient and staff safety stemming from inadequate equipment maintenance and improper sterilization practices. Reported concerns included the transmission of infections, accidental burns from overheating light cables, and intraoperative delays caused by equipment malfunctions.

When equipment suddenly stops working during surgery, everyone in the OT becomes tense because if troubleshooting takes too much time, it can affect patient safety. (Resident 6)

Sometimes the light cable becomes very hot during long surgeries, and if it is not handled carefully, there is a chance of burns to staff or even the patient; this makes the situation very scary. (OT Technician 7)

Even a small mistake during cleaning or sterilization of laparoscopic instruments can increase infection risk for the patient; this puts us under extra pressure. (Nursing Officer 1)

Detailed thematic findings and representative observations are summarized in Table 2.

**Table 2:** Major themes identified during FGDs SOP: standard operating procedure, CO₂: carbon dioxide, OT: operating theater, FGDs: focus group discussions

Theme	Representative findings
Training deficiencies	Lack of structured workshops, simulation-based training, and formal SOP orientation
Equipment-related challenges	Fogging, overheating cables, monitor shortages, CO₂ leakage
Sterilization and reprocessing issues	Improper drying before sterilization and reuse of disposable instruments
Instrument handling difficulties	Difficulty identifying and assembling laparoscopic instruments
Safety concerns	Risk of infection transmission, equipment malfunction, and intraoperative delays
Workflow and communication barriers	Delays in OT setup and communication gaps among OT personnel

Subgroup analysis demonstrated distinct perspectives among the three participant groups. OT technicians predominantly discussed technical and maintenance-related concerns, including damage to delicate cables, preventive servicing requirements, and sterilization limitations. Nursing officers mainly emphasized workflow coordination, patient safety concerns, and the need for standardized checklists. Residents primarily highlighted deficiencies in troubleshooting skills and formal laparoscopic training. The subgroup-specific concerns and suggested improvements reported by participants are summarized in Table 3.

**Table 3:** Subgroup-specific findings and recommendations OT: operating theater

Participant group	Predominant concerns	Suggested improvements
OT technicians	Maintenance burden, sterilization limitations, equipment malfunction	Preventive maintenance schedules and dedicated servicing support
Nursing officers	Checklist deficiencies, workflow delays, communication gaps	Regular workshops and standardized preoperative checklists
Residents	Troubleshooting difficulties and inadequate training	Simulation-based learning and structured orientation programs

Overall, the findings suggest that laparoscopic instrument maintenance challenges are multifactorial and closely linked to inadequate training opportunities, equipment limitations, sterilization-related concerns, and workflow inefficiencies. Participants across all groups recommended institution-based standard operating procedures, preventive maintenance schedules, regular multidisciplinary workshops, and simulation-based competency training programs to strengthen perioperative safety and improve operative efficiency.

## Discussion

The present qualitative study explored the experiences, operational challenges, safety concerns, and training needs related to laparoscopic instrument maintenance among OT technicians, nursing officers, and residents working in the Department of Obstetrics and Gynecology. The findings demonstrated that laparoscopic instrument management is a multidimensional process influenced by inadequate training, equipment-related limitations, sterilization challenges, workflow barriers, and patient safety concerns. Training deficiencies emerged as one of the most prominent concerns across all participant groups, highlighting the need for structured educational interventions and standardized institutional protocols. Similar challenges have been reported in other low- and middle-income countries, suggesting a broader systemic issue [4].

Most participants reported learning laparoscopic troubleshooting informally during routine surgical procedures rather than through structured educational programs. Residents particularly expressed a lack of confidence in independently assembling laparoscopic systems and managing equipment-related technical issues. Similar findings have been reported by Wilkinson et al. (2021), who identified inadequate infrastructure, a shortage of trained personnel, and limited access to simulation-based training as major barriers to laparoscopic surgery programs in low- and middle-income countries [4]. Likewise, Özkan et al. (2025) emphasized that structured competency-based training and technology-oriented educational curricula are essential for improving perioperative efficiency and patient safety in minimally invasive surgery [10].

The present study also identified several equipment-related challenges, including fogging of camera lenses, overheating of light source cables, malfunctioning insufflator tubing, monitor shortages, and CO₂ leakage from reused trocars. Participants perceived these technical problems as important contributors to operative delays, workflow interruptions, and intraoperative stress among OT personnel. These findings are consistent with observations reported by Madhok et al. (2022), who highlighted that inadequate maintenance and equipment malfunction may compromise laparoscopic safety and increase the risk of technical complications during surgery [11]. Similarly, Ilkaz and Gençbaş (2025) reported that OT nurses considered laparoscopic surgery technically demanding because of equipment complexity, ergonomic burden, and technology-related stress [8].

Sterilization and reprocessing issues emerged as another important theme. Participants reported challenges related to improper drying of instruments before hydrogen peroxide gas plasma sterilization (e.g., STERRAD), inadequate cleaning of internal channels, and reuse of disposable laparoscopic accessories due to resource limitations. These concerns are clinically significant because inadequate sterilization may increase the risk of surgical site infections, compromise patient safety, and reduce instrument longevity. Robertson et al. (2021) similarly identified deficiencies in laparoscopic instrument reprocessing and lack of standardized sterilization infrastructure in resource-limited healthcare settings [3].

Workflow inefficiencies and communication barriers were also frequently discussed by participants, particularly nursing officers and residents. Delays in emergency laparoscopic setup, absence of standardized preoperative checklists, and communication gaps between surgeons and OT staff were perceived to negatively affect teamwork and operative efficiency. The clinical consequences of communication failures in the OT are well illustrated by cases of retained surgical foreign bodies (gossypibomas), where inadequate communication and counting errors have led to serious patient harm and medicolegal consequences [12]. This underscores the need for robust communication protocols and standardized checklists to prevent errors, regardless of the perceived accuracy of routine counts. McMullan et al. (2021) demonstrated that OT interruptions, workflow disruptions, and communication failures are associated with increased technical errors and compromised patient safety outcomes [5]. These findings highlight the importance of multidisciplinary coordination and checklist-based perioperative approaches in minimally invasive surgery.

An important strength of the present study is the inclusion of multiple categories of healthcare professionals directly involved in laparoscopic surgeries, including residents, nursing officers, and OT technicians. This multidisciplinary representation enabled triangulation of perspectives, enhancing the credibility of the findings and facilitating a comprehensive exploration of operational and safety-related concerns across the OT environment. Furthermore, the qualitative design facilitated in-depth understanding of practical challenges and contextual experiences that are often overlooked in quantitative assessments.

However, certain limitations should be acknowledged. The study was conducted in a single tertiary care teaching institution, which may limit transferability to other healthcare settings. However, the findings may still be relevant to similar tertiary-care teaching hospitals in India, where laparoscopic practices and training structures are comparable. The qualitative design also limits the ability to establish causal relationships or quantify the impact of specific maintenance challenges on surgical outcomes. Additionally, participant responses may have been influenced by recall bias or social desirability bias during FGDs. Direct observational assessment of laparoscopic instrument handling practices was not performed in the present study. Future multicentric mixed-methods studies incorporating observational audits and quantitative outcome measures may provide broader evidence regarding laparoscopic instrument maintenance and patient safety practices.

The findings of the present study have important practical implications for improving perioperative safety and quality in minimally invasive gynecological surgery. Institution-based standard operating procedures, preventive maintenance schedules, regular equipment audits, and simulation-based competency training programs may significantly improve laparoscopic instrument management practices. Additionally, multidisciplinary workshops involving surgeons, nursing officers, and OT technicians may strengthen teamwork, communication, and troubleshooting efficiency in the OT.

## Conclusions

The present study highlights that laparoscopic instrument maintenance is an important yet often underrecognized component of minimally invasive surgical safety and requires a systems-level approach that includes institutional protocols, training, and adequate resource allocation. Challenges related to inadequate training, equipment malfunctions, sterilization practices, and workflow coordination were found to affect operative efficiency and perioperative patient safety. Participants emphasized the need for competency-based training, multidisciplinary teamwork, standardized maintenance protocols, preventive service schedules, and simulation-based learning programs to improve laparoscopic instrument handling.
